# Associations Between Hygiene- and Nutrition-Related Practices and Pregnancy Outcomes: A Cross-Sectional Study

**DOI:** 10.3390/medicina62091676

**Published:** 2026-08-31

**Authors:** Alin Vasile Kadaș, Radu Neamțu, Roxana Mariana Chiș, Cezar Pântea, Olga Pântea, Oana Neda-Stepan, Adrian Gluhovschi

**Affiliations:** 1 Medicine Faculty, Victor Babeș University of Medicine and Pharmacy, 300041 Timisoara, Romania; alin.kadas@umft.ro (A.V.K.);; 2 Faculty of Educational Sciences Psychology and Social Sciences, Aurel Vlaicu University of Arad, 310032 Arad, Romania; 3 Obstetrics and Gynecology Department II, County Emergency Hospital Timisoara, 300723 Timisoara, Romania; 4 Plastic Surgery Department, County Emergency Hospital Timisoara, 300723 Timisoara, Romania; olgafurtuna11@gmail.com

**Keywords:** pregnancy, hygiene, nutrition, supplementation, vaginal candidiasis, urinary tract infection, pregnancy outcome, pregnancy complications

## Abstract

*Background and Objectives*: Pregnancy is accompanied by physiological, hormonal, immunological, and metabolic changes that may increase susceptibility to infections and adverse maternal or fetal outcomes. This study aimed to evaluate hygiene- and nutrition-related behaviors among pregnant or previously pregnant women and to assess their associations with infections, pregnancy complications, and pregnancy outcomes. *Materials and Methods*: An observational, descriptive, and analytical cross-sectional study was conducted using an anonymous questionnaire administered to women aged 18–40 years from Timiș County, Romania, who were currently pregnant or had experienced at least one previous pregnancy. A total of 361 participants were included in the final analysis. Descriptive and bivariate analyses were performed, followed by multivariable binary logistic regression. Multicollinearity was assessed using variance inflation factors, and Firth’s penalized logistic regression was performed as a sensitivity analysis. *Results*: In multivariable analyses, occasional intimate hygiene, compared with twice-daily intimate hygiene, was associated with higher odds of vaginal candidiasis (aOR = 6.63; 95% CI: 2.29–19.17) and urinary tract infection (aOR = 4.36; 95% CI: 1.73–10.99). An unbalanced diet was associated with higher odds of pregnancy complications compared with a balanced diet (aOR = 2.33; 95% CI: 1.26–4.30). Compared with women reporting three or more supplements, women reporting no supplementation had markedly higher odds of an adverse pregnancy outcome (aOR = 46.15; 95% CI: 20.45–104.15), while each additional previous abortion was associated with higher odds of an adverse outcome (aOR = 2.25; 95% CI: 1.41–3.59). Firth sensitivity analysis attenuated the estimate for no supplementation (OR = 37.54; 95% CI: 17.89–85.32) but did not materially change the principal findings. *Conclusions*: Intimate hygiene frequency, nutritional supplementation, perceived diet quality, and reproductive history were associated with pregnancy-related outcomes in this study. These findings support structured prenatal assessment and counselling regarding nutrition, evidence-based supplementation, reproductive history, and appropriate hygiene practices. Because of the cross-sectional observational design and potential residual confounding, the findings should be interpreted as associations rather than causal effects.

## 1. Introduction

Pregnancy represents a complex physiological period in life, which is defined by anatomic, hormonal and immunological changes in the organism of a woman. During pregnancy, energy and nutrient demands increase to support maternal metabolic adaptations, including the expansion of blood volume and red blood cell mass, as well as to ensure an adequate supply of energy and essential nutrients to the developing fetus. In this specific time span, women are far more susceptible to certain infections and metabolic disorders, hence the need for adequate changes in hygiene and nutrition, for maintaining a proper evolution of the pregnancy. Promoting correct personal hygiene habits and a balanced diet are essential elements in preventing maternal and fetal complications and ensuring optimal fetal development [1,2].

According to World Health Organization recommendations on maternal health, antenatal care should include clinical monitoring of pregnancy and education of the pregnant woman on personal hygiene, nutrition and infection prevention. Appropriate hygiene practices, such as proper hand washing, maintaining oral hygiene and intimate hygiene, contribute to reducing the risk of urinary, vaginal or systemic infections, which are frequently encountered during pregnancy [3]. At the same time, appropriate nutritional supplementation and the adoption of balanced eating habits have an important role in preventing anemia, micronutrient deficiencies and other associated complications. FIGO (International Federation of Gynecology and Obstetrics) recommends daily multiple-micronutrient supplementation during pregnancy, together with individualized nutritional counseling when needed. Key recommendations include iron and folic acid supplementation, iodine intake, cautious use of vitamin A, and routine prenatal doses of vitamin D, while calcium and vitamin E supplementation are not clearly supported for improving neonatal outcomes. Overall, supplementation should be adapted to maternal needs and used within the recommended daily limits [4].

The physiological vaginal microbiota of pregnant women plays a crucial role in the natural defense against infections. Alterations in this microbiological balance have been shown to significantly influence pregnancy outcomes, being associated with complications such as premature rupture of membranes and preterm birth. Moreover, disruption of the maternal vaginal flora may contribute to an imbalance in the neonatal intestinal microbiome, which can have long-term effects on neurological, respiratory, metabolic, and immunological development, as well as potentially increasing neonatal morbidity and mortality [5]. Preventing infections during pregnancy should be prioritized above treating them, considering these consequences. Comprehensive medical history, ongoing immunization status monitoring and updating, and, when necessary, targeted serological studies to determine infectious status are all necessary for effective prophylaxis. The gynecologist might offer tailored advice regarding suitable preventive measures based on these evaluations. The current strategy emphasizes how crucial it is to put guidelines into practice for the management and prevention of infections that are essential to obstetrics during pregnancy. Maternal infections during pregnancy, such as urinary tract infections, vaginal candidiasis, toxoplasmosis or viral infections, can negatively influence the course of pregnancy and may be associated with premature birth, intrauterine growth restriction or other adverse obstetric outcomes. In this context, hygiene behaviors and lifestyle adopted by the pregnant woman become important factors that can contribute to reducing risks and improving maternal and fetal prognosis [6].

Essential vitamins and minerals are among the micronutrients that are crucial for sustaining maternal health and promoting fetal growth and development throughout pregnancy. Both the mother and the growing fetus may suffer from deficiencies in essential nutrients, which could result in several issues. Maternal anemia, which is frequently linked to iron deficiency, may raise the risk of death from hemorrhage after childbirth. Its precise effect on fetal development and pregnancy outcomes is still unknown, though. Although its relationship with other unfavorable birth outcomes is still undetermined, folic acid deficiency is known to have significant hematological effects and has been connected to pregnancy problems and congenital abnormalities. Zinc deficiency has been correlated in certain studies with complications during pregnancy and delivery, as well as with intrauterine growth restriction, congenital anomalies, and delayed neurobehavioral and immunological development in the fetus, though findings are not entirely consistent across all research. Iodine deficiency during pregnancy represents a significant public health concern, as it may lead to severe outcomes such as cretinism, increased risk of fetal loss, and preterm birth. Additionally, insufficient levels of other minerals, including magnesium, selenium, copper, and calcium, have been associated with various complications related to pregnancy, labor, and fetal development. Deficiencies in vitamins other than folic acid may similarly contribute to adverse maternal and fetal outcomes, highlighting the importance of adequate micronutrient intake throughout pregnancy [7].

The aim of this study is to describe hygiene and nutrition behaviors among women aged 18 to 40 years in Timis County Romania and to evaluate the possible association between these behaviors and pregnancy outcome, including the occurrence of complications or infections during gestation. This study aims to highlight the importance of adopting adequate hygiene and nutrition measures during pregnancy to maintain maternal and fetal health.

## 2. Materials and Methods

### 2.1. Study Design and Participants

The present study was designed as an observational, descriptive, and analytical cross-sectional study conducted among women attending the emergency care unit and outpatient clinic of the County Emergency Hospital Timisoara, Romania. Eligible participants were women aged 18–40 years, residents of Timiș County, who were currently pregnant or had experienced at least one previous pregnancy ending in birth or spontaneous abortion and who provided informed consent. Exclusion criteria were age outside the predefined range, residence outside Timiș County, absence of a current or previous pregnancy, refusal to provide informed consent, and incomplete or inadequately completed questionnaires. Participation was voluntary and anonymous, and no personally identifiable information was collected.

The questionnaire was provided to eligible participants during their presentation at the clinic after they expressed willingness to participate, and informed consent was required before questionnaire completion. The recruitment target was approximately 400 completed questionnaires. After eligibility screening and data cleaning, the final analytical sample comprised 361 participants.

### 2.2. Questionnaire and Study Variables

Data was collected using an author-developed 16-item online questionnaire administered through Google Forms. The questionnaire assessed sociodemographic characteristics, reproductive history, hand, oral and intimate hygiene, nutritional supplementation, perceived diet quality, vaccination status, diagnosed infections, pregnancy-related complications, and the outcome of the most recent pregnancy. Hygiene and nutritional behaviors were self-reported. The complete questionnaire and response options are provided in Supplementary File S1.

The outcome of the most recent pregnancy was categorized as term birth, preterm birth (<37 completed weeks of gestation), post-term pregnancy (>41 completed weeks, corresponding to ≥42 weeks of gestation), or spontaneous abortion. Gestational age at spontaneous abortion was not collected separately. Pregnancy-related infections and complications included urinary tract infection, vaginal candidiasis, bacterial vaginosis, influenza, cytomegalovirus infection, toxoplasmosis, anemia, gestational diabetes, and hypertensive disorders of pregnancy.

### 2.3. Data Management and Variable Coding

The dataset was checked for internal consistency before statistical analysis, particularly for the reported numbers of pregnancies, births, and spontaneous abortions. Corrections were made only when the remaining response pattern allowed for an unambiguous interpretation. The cleaning log contained 16 corrections across nine participant records, restricted to reproductive-history count variables. No pregnancy-outcome, hygiene, dietary, supplementation, infection, or complication categories were modified during data cleaning.

For statistical analysis, pregnancy outcome was dichotomized as term birth versus adverse pregnancy outcome. The composite adverse pregnancy outcome included spontaneous abortion, preterm birth, and post-term pregnancy, while its individual components were reported separately in the descriptive analysis. Because the questionnaire did not collect the timing of supplement initiation relative to spontaneous abortion, the temporal sequence between supplementation and abortion could not be established for these cases. To address potential reverse causality, a sensitivity analysis was therefore performed excluding all participants whose most recent pregnancy ended in spontaneous abortion. In this analysis, the adverse pregnancy outcome comprised preterm and post-term birth, with term birth as the reference outcome. Nutritional supplementation was grouped as no supplements, 1–2 supplements, or ≥3 supplements. Diagnosed infection and pregnancy complications or infections were coded as binary variables. Separate binary outcomes were created for urinary tract infection and vaginal candidiasis. Vaccination status was categorized as vaccinated or not vaccinated/unknown.

### 2.4. Statistical Analysis

Statistical analyses were performed using Python version 3.13.5 (Python Software Foundation, Wilmington, DE, USA), SciPy version 1.17.0, and statsmodels version 0.14.6. Categorical variables were summarized as frequencies and percentages. Associations between categorical variables were assessed using the Chi-square test or Fisher’s exact test when expected cell frequencies were small, while the Mann–Whitney U test was used for numerical count variables when appropriate. Bonferroni correction was applied separately to the six predefined main bivariate analyses and the three secondary analyses, corresponding to corrected significance thresholds of *p* < 0.0083 and *p* < 0.0167, respectively.

Five multivariable binary logistic regression models were constructed for adverse pregnancy outcome, pregnancy complications, diagnosed infection, vaginal candidiasis, and urinary tract infection. Predictors were selected according to clinical relevance, conceptual relationship with the respective outcome, and potential confounding rather than automated stepwise selection. The results were expressed as adjusted odds ratios (aORs), 95% confidence intervals (CIs), and *p*-values. Multicollinearity was assessed using variance inflation factors (VIFs), with VIF > 5 considered potentially problematic. Firth’s penalized logistic regression was additionally performed as a sensitivity analysis because some outcomes involved relatively small numbers of events and large conventional odds-ratio estimates. Complete coefficient estimates for all variables entered the conventional and Firth penalized logistic regression models are provided in Supplementary File S3. As an additional sensitivity analysis, Model 1 was repeated after excluding participants with a reported history of spontaneous abortion (number of previous abortions ≥ 1). Because previous abortion count was constant at zero in the restricted sample, this variable was omitted from the sensitivity model, while the remaining predictor structure was retained. Model discrimination was assessed using the area under the receiver operating characteristic curve (AUC). Calibration was evaluated graphically by comparing observed outcome frequencies with mean predicted probabilities across quintiles of predicted risk. The number of observations included in each model was also recorded after accounting for missing data. No missing values were present for the variables included in the five multivariable models; therefore, all models were estimated using the complete analytical sample of 361 participants.

For the adverse pregnancy outcome model, an additional sensitivity analysis was conducted after excluding all spontaneous-abortion outcomes to reduce the potential for reverse causality related to the unknown timing of supplement initiation. The same predictor structure as in the primary Model 1 was retained. Additional pregnancy-outcome sensitivity analyses excluded currently pregnant participants to improve temporal alignment between exposures and outcome; Model 1 was also repeated without previous abortion count because of its potential overlap with the composite adverse pregnancy outcome.

Pre-pregnancy and gestational BMI, socioeconomic status, smoking status, gestational age at questionnaire completion, comprehensive maternal comorbidity data, and detailed information on supplement dose and duration were not collected and therefore could not be included as covariates in the multivariable analyses. A two-sided *p*-value < 0.05 was considered statistically significant in the multivariable analyses. All findings were interpreted as associations rather than causal effects because of the observational cross-sectional design.

### 2.5. Ethical Considerations

This study respected the ethical principles of scientific research, and by completing the questionnaire, the participants expressed their consent for the use of the data for academic and scientific purposes. Approval was obtained from the Ethical committee of the Emergency Hospital of Timis County (No. 260/16 July 2025).

## 3. Results

A total of 361 participants were included in the analysis. Sociodemographic and reproductive characteristics are presented in Table 1. Age group, residence, and educational level did not differ significantly according to pregnancy outcome. Reproductive status differed between outcome groups (*p* = 0.012): the category “previous spontaneous abortion” was approximately 1.9-fold more frequent in the adverse-outcome group than in the term-outcome group (15.5% vs. 8.3%), while current pregnancy status was approximately 1.4-fold more frequent (35.1% vs. 25.8%); conversely, a previous pregnancy ending in birth was less frequent in the adverse-outcome group (49.5% vs. 65.9%). Previous abortion history also differed between groups (*p* < 0.001), with one previous abortion reported approximately twice as frequently in the adverse-outcome group compared to the term-outcome group (68.0% vs. 34.1%). In an additional sensitivity analysis, Model 1 was repeated after excluding women with a reported history of spontaneous abortion. The restricted analysis included 186 participants, of whom 25 had an adverse pregnancy outcome. Because the previous abortion count was zero for all participants in this analysis, this variable was omitted from the model. Compared with women reporting three or more supplements, those reporting no supplementation continued to have substantially higher adjusted odds of an adverse pregnancy outcome (aOR = 24.75; 95% CI: 6.97–87.85; *p* < 0.001). For women reporting 1–2 supplements, the direction of the association was unchanged, although the estimate was less precise and no longer statistically significant (aOR = 2.74; 95% CI: 0.71–10.65; *p* = 0.145).

Obstetrics and medical characteristics are presented in Table 2. Term delivery was the most frequently reported outcome (*n* = 264; 73.1%), whereas 97 participants (26.9%) reported an adverse pregnancy outcome. Pregnancy complications or infections were reported by 173 participants (47.9%). In the exploratory comparison according to pregnancy outcome, pregnancy complications or infections were approximately 1.27-fold more frequent in the adverse-outcome group than in the term-outcome group (56.7% vs. 44.7%; χ^2^ = 4.10; df = 1; *p* = 0.043). Diagnosed infections were also numerically more frequent in the adverse-outcome group (32.0% vs. 22.7%; approximately 1.41-fold), although this difference was not statistically significant (χ^2^ = 3.21; df = 1; *p* = 0.073). Vaccination frequencies were nearly identical between groups (5.2% vs. 5.3%; Fisher’s exact test, *p* = 1.000).

Hygiene- and nutrition-related behaviors according to pregnancy outcome are presented in Table 3. Supplementation showed the largest difference between groups: no supplementation was approximately 12.2-fold more frequent in the adverse-outcome group than in the term-outcome group (59.8% vs. 4.9%), whereas use of three or more supplements was approximately 3.3-fold more frequent in the term-outcome group (78.8% vs. 23.7%; *p* < 0.001). Occasional intimate hygiene was approximately 1.9-fold more frequent in the adverse-outcome group (24.7% vs. 13.3%; *p* = 0.032), and an unbalanced diet was approximately 1.7-fold more frequent (27.8% vs. 16.3%; *p* = 0.041). Hand and oral hygiene did not differ significantly between outcome groups.

Bivariate analyses were performed to evaluate the association between hygiene- and nutrition-related behaviors and pregnancy-related outcomes. The main bivariate analyses included six predefined comparisons: intimate hygiene and vaginal candidiasis, hand hygiene and urinary tract infection, intimate hygiene and urinary tract infection, oral hygiene and diagnosed infection, diet quality and adverse pregnancy outcome, and supplementation group and pregnancy outcome. Since multiple comparisons were performed, Bonferroni correction was applied to this group of analyses, resulting in a corrected significance threshold of *p* < 0.0083. The results can be visualized in Table 4.

A statistically significant association was observed between intimate hygiene frequency and vaginal candidiasis (χ^2^ = 46.10; df = 2; raw *p* < 0.001; Bonferroni-adjusted *p* < 0.001). Vaginal candidiasis occurred in 27.1% of women reporting occasional intimate hygiene compared with 5.3% of those reporting intimate hygiene twice daily, corresponding to an approximately 5.2-fold difference. Although one expected cell count was slightly below 5, the strength of the association supports the relevance of this finding, which should nevertheless be interpreted with caution.

The association between hand hygiene frequency and urinary tract infection was not statistically significant (χ^2^ = 5.71; df = 2; raw *p* = 0.058; Bonferroni-adjusted *p* = 0.345). In contrast, intimate hygiene frequency was significantly associated with urinary tract infection (χ^2^ = 11.48; df = 2; raw *p* = 0.003; Bonferroni-adjusted *p* = 0.019). Urinary tract infection occurred in 27.1% of women reporting occasional intimate hygiene compared with 7.9% of those reporting intimate hygiene twice daily, corresponding to an approximately 3.4-fold difference.

No significant association was identified between oral hygiene frequency and diagnosed infections during pregnancy (χ^2^ = 2.35; df = 2; raw *p* = 0.309; Bonferroni-adjusted *p* = 1.000). Diet quality was associated with adverse pregnancy outcome at the conventional significance level (χ^2^ = 6.38; df = 2; raw *p* = 0.041), with adverse outcomes occurring in 38.6% of women reporting an unbalanced diet compared with 22.5% of those reporting a balanced diet, corresponding to an approximately 1.7-fold difference; however, this association did not remain significant after Bonferroni correction (adjusted *p* = 0.247). The supplementation group showed a strong association with pregnancy outcome (χ^2^ = 142.22; df = 2; raw *p* < 0.001; Bonferroni-adjusted *p* < 0.001), with adverse outcomes occurring in 81.7% of women reporting no supplementation compared with 10.0% of women reporting three or more supplements, corresponding to an approximately 8.2-fold difference.

Secondary bivariate analyses were also performed for vaccination status, educational level, and age group as presented in Table 5. For these three secondary comparisons, Bonferroni correction resulted in a corrected significance threshold of *p* < 0.0167. Vaccination status was not significantly associated with diagnosed infections during pregnancy (Fisher’s exact test, raw *p* = 0.424; adjusted *p* = 1.000). Educational level was not significantly associated with supplementation group (χ^2^ = 7.61; df = 4; raw *p* = 0.107; adjusted *p* = 0.321), and age group was not significantly associated with adverse pregnancy outcome (χ^2^ = 3.72; df = 3; raw *p* = 0.294; adjusted *p* = 0.882).

Multivariable binary logistic regression models were performed to identify factors independently associated with adverse pregnancy outcome, pregnancy complications, diagnosed infections, vaginal candidiasis, and urinary tract infection. The results are reported as adjusted odds ratios (aORs) with 95% confidence intervals (95% CIs). These models were used to determine whether the observed bivariate associations remained present after adjustment for relevant sociodemographic, nutritional, hygiene-related, and reproductive variables. Multicollinearity assessment showed no evidence of problematic collinearity among the predictors included in the multivariable models. VIF values ranged from 1.03 to 2.65 across the five models, with all values remaining below the predefined threshold of 5. The principal estimates from the five multivariable logistic regression models are summarized in Table 6.

In the first model, adverse pregnancy outcome was used as the dependent variable, with term birth as the reference category. The model included supplementation group, diet quality, number of previous abortions, age group, educational level, and residence. After adjustment, the supplementation group remained strongly associated with adverse pregnancy outcome. Compared with women who used three or more supplements, women using 1–2 supplements had higher odds of adverse outcome (aOR = 3.32; 95% CI: 1.52–7.21; *p* = 0.002), while women reporting no supplementation had markedly increased odds of adverse outcome (aOR = 46.15; 95% CI: 20.45–104.15; *p* < 0.001). To address potential reverse causality related to the unknown timing of supplement initiation, Model 1 was repeated after excluding all 55 participants whose most recent pregnancy ended in spontaneous abortion. The sensitivity analysis therefore included 306 participants, with 42 adverse outcomes comprising preterm and post-term births. Compared with women reporting three or more supplements, women reporting 1–2 supplements had higher adjusted odds of adverse outcome (aOR = 2.83; 95% CI: 1.07–7.47; *p* = 0.036), while those reporting no supplementation had markedly higher adjusted odds (aOR = 26.23; 95% CI: 9.88–69.66; *p* < 0.001). Thus, the association between supplementation level and adverse pregnancy outcome remained present after spontaneous-abortion cases were excluded, although the magnitude of the estimates was attenuated. The number of previous abortions was also independently associated with adverse pregnancy outcome (aOR = 2.25; 95% CI: 1.41–3.59; *p* < 0.001). Unbalanced diet showed a trend toward increased odds of adverse outcome, but this did not reach statistical significance after adjustment (aOR = 2.11; 95% CI: 0.90–4.94; *p* = 0.085). Additional sensitivity analyses supported the main Model 1 findings. After excluding the 102 participants who were currently pregnant, the restricted sample included 259 participants with 63 adverse outcomes; the supplementation association remained significant, with aORs of 4.42 (95% CI: 1.64–11.92; *p* = 0.003) for 1–2 supplements and 78.23 (95% CI: 25.23–242.63; *p* < 0.001) for no supplementation. When Model 1 was repeated without previous abortion count, the corresponding estimates remained similar (aOR = 3.20; 95% CI: 1.51–6.77; *p* = 0.002, and aOR = 46.55; 95% CI: 20.95–103.42; *p* < 0.001, respectively).

The second model evaluated predictors of pregnancy complications. The dependent variable was the presence of at least one complication or infection during pregnancy. The model included diet quality, supplementation group, intimate hygiene, oral hygiene, age group, educational level, residence, and total number of pregnancies. Compared with women reporting a balanced diet, those reporting an unbalanced diet had significantly higher odds of pregnancy complications (aOR = 2.33; 95% CI: 1.26–4.30; *p* = 0.007). Relatively balanced diet showed a borderline association with complications compared with balanced diet (aOR = 1.63; 95% CI: 0.99–2.66; *p* = 0.052). Occasional intimate hygiene was also associated with increased odds of pregnancy complications compared with intimate hygiene twice daily (aOR = 2.39; 95% CI: 1.21–4.72; *p* = 0.012).

The third model assessed predictors of diagnosed infections during pregnancy. The model included intimate hygiene, hand hygiene, diet quality, supplementation group, vaccination status, educational level, residence, and age group. Occasional intimate hygiene remained strongly associated with diagnosed infections after adjustment (aOR = 5.01; 95% CI: 2.34–10.71; *p* < 0.001). Hand hygiene and vaccination status were not independently associated with diagnosed infection. Vaccination showed no statistically significant association with infection risk in this model (aOR = 0.59; 95% CI: 0.16–2.14; *p* = 0.421).

The fourth model examined predictors of vaginal candidiasis. The model included intimate hygiene, diet quality, educational level, and age group. Occasional intimate hygiene remained independently associated with vaginal candidiasis. Compared with women reporting intimate hygiene twice daily, those reporting occasional intimate hygiene had significantly higher odds of vaginal candidiasis (aOR = 6.63; 95% CI: 2.29–19.17; *p* < 0.001). Once-daily intimate hygiene was not significantly associated with candidiasis compared with twice-daily intimate hygiene (aOR = 0.31; 95% CI: 0.07–1.28; *p* = 0.105).

The fifth model evaluated predictors of urinary tract infection. The model included intimate hygiene, hand hygiene, age group, educational level, and total number of pregnancies. Intimate hygiene remained independently associated with urinary tract infection. Compared with women reporting intimate hygiene twice daily, those reporting once-daily intimate hygiene had increased odds of urinary tract infection (aOR = 2.35; 95% CI: 1.04–5.29; *p* = 0.040), while those reporting occasional intimate hygiene had even higher odds (aOR = 4.36; 95% CI: 1.73–10.99; *p* = 0.002). The total number of pregnancies was also independently associated with urinary tract infection (aOR = 1.46; 95% CI: 1.08–1.98; *p* = 0.013). Hand hygiene was not independently associated with urinary tract infection after adjustment.

To assess the robustness of the conventional logistic regression estimates, Firth’s penalized logistic regression was performed as a sensitivity analysis for all five models (Table 7). The direction and statistical interpretation of the principal associations remained materially unchanged. In Model 1, the association between no supplementation and adverse pregnancy outcome was attenuated from an aOR of 46.15 in conventional logistic regression to a Firth OR of 37.54 (95% CI: 17.89–85.32; *p* < 0.001). The use of 1–2 supplements versus three or more supplements also remained associated with adverse pregnancy outcome (Firth OR = 3.16; 95% CI: 1.48–6.73; *p* = 0.003), as did the number of previous abortions (Firth OR = 2.16; 95% CI: 1.38–3.43; *p* < 0.001). Similar stability was observed in the remaining models: unbalanced diet remained associated with pregnancy complications (Firth OR = 2.23; 95% CI: 1.23–4.10; *p* = 0.008), and occasional intimate hygiene remained associated with diagnosed infection (Firth OR = 4.59; 95% CI: 2.22–9.75; *p* < 0.001), vaginal candidiasis (Firth OR = 5.73; 95% CI: 2.18–16.77; *p* < 0.001), and urinary tract infection (Firth OR = 4.04; 95% CI: 1.68–10.20; *p* = 0.002). These sensitivity analyses indicate that the principal findings were robust to penalized-likelihood estimation. Complete conventional and Firth regression estimates for all variables entered into the five models are provided in Supplementary File S3.

All five multivariable models included the complete analytical sample of 361 participants because no missing values were present in the variables entered into the respective models. AUC values ranged from 0.650 to 0.862, with the highest values observed for the adverse pregnancy outcome model (AUC = 0.862) and the vaginal candidiasis model (AUC = 0.832). Calibration plots for all five models are provided in Supplementary File S2.

Overall, the multivariable analyses supported the independent association of supplementation level and previous abortion history with adverse pregnancy outcome, diet quality with pregnancy complications, and intimate hygiene with diagnosed infections, vaginal candidiasis, and urinary tract infection. These findings should be interpreted as associations rather than causal effects given the observational and cross-sectional design of this study.

## 4. Discussion

The main findings were associations of supplementation level with adverse pregnancy outcome, diet quality with pregnancy complications, and intimate hygiene frequency with vaginal candidiasis and urinary tract infection. Hand hygiene, oral hygiene, vaccination status, educational level, and age were not consistently associated with the studied outcomes. These findings suggest outcome-specific associations rather than a general effect of hygiene practices. Previous literature has emphasized the importance of hygiene counselling and infection-prevention behaviors during pregnancy [5,8]; however, these sources addressed broader preventive practices and different infectious outcomes. The following discussion therefore focuses on the specific associations identified in the present study.

One of the most relevant findings of this study was the association between intimate hygiene and vaginal candidiasis. In the conventional multivariable logistic regression model, women reporting occasional intimate hygiene had 6.63-fold higher adjusted odds of vaginal candidiasis compared with women reporting intimate hygiene twice daily (95% CI: 2.29–19.17). The association remained strong in the Firth sensitivity analysis (OR = 5.73; 95% CI: 2.18–16.77), indicating that the magnitude and direction of the association were not substantially altered by penalized estimation.

These findings are biologically plausible in the context of the vaginal microbiome. Holdcroft et al. [9] emphasized that intimate hygiene practices can influence the vaginal microbial environment and summarized previous studies in which vaginal douching was associated with increased risks of adverse reproductive and genital outcomes, with reported effect estimates generally in the range of approximately two- to four-fold for some outcomes. The magnitude observed in the present study for vaginal candidiasis was therefore comparatively large. However, direct quantitative comparison is limited because the studies reviewed by Holdcroft et al. evaluated different intimate hygiene practices and different clinical outcomes. Moreover, the present questionnaire assessed the frequency of intimate hygiene but did not specifically evaluate vaginal douching, the type of hygiene products used, or the direction and technique of washing. Consequently, the observed association should not be interpreted as evidence that more frequent intimate washing itself prevents candidiasis, but rather as an association requiring confirmation in studies with more detailed assessment of intimate hygiene practices.

Intimate hygiene was also associated with urinary tract infection. In the conventional multivariable model, compared with women reporting intimate hygiene twice daily, those reporting once-daily intimate hygiene had 2.35-fold higher adjusted odds of urinary tract infection (95% CI: 1.04–5.29), while those reporting occasional intimate hygiene had 4.36-fold higher adjusted odds (95% CI: 1.73–10.99). The association for occasional intimate hygiene remained similar in the Firth sensitivity analysis (OR = 4.04; 95% CI: 1.68–10.20). In addition, each additional pregnancy was associated with increased odds of urinary tract infection (aOR = 1.46; 95% CI: 1.08–1.98). These findings are broadly consistent with the work of Dimetry et al. [10], who reported significant associations of urinary tract infection during pregnancy with unsatisfactory personal hygiene and higher gravidity, among other socio-demographic and reproductive factors. However, direct comparison of effect sizes is not possible because the exposures, study design, and statistical modeling differed between the two studies; notably, low socioeconomic status emerged as the independent predictor of urinary tract infection in the multivariable analysis reported by Dimetry et al. The present findings should therefore be interpreted as evidence of an association rather than a causal effect of hygiene frequency itself. Urinary tract infections during pregnancy remain clinically relevant because of their association with adverse maternal and pregnancy outcomes, including preterm delivery and low birth weight [10].

Maternal infections can also affect pregnancy through systemic inflammatory pathways. Cline et al. emphasized the importance of diagnosing and managing maternal infections during pregnancy [6]. In addition, Weckman et al. described how maternal infection and the inflammatory response may interfere with placental vascular development, including vasculogenesis and angiogenesis, thereby contributing to altered placental hemodynamics and adverse birth outcomes such as preterm delivery, small-for-gestational-age infants, stillbirth, and low birth weight [11]. These mechanisms provide biological plausibility for the relationship between maternal infection and unfavorable pregnancy outcomes.

Regarding nutrition, supplementation level was strongly associated with pregnancy outcome. In the bivariate analysis, adverse pregnancy outcomes were considerably more frequent among women reporting no supplementation, while term birth was more common among women using three or more supplements. This association remained strong in the multivariable logistic regression model after adjustment for diet quality, number of previous abortions, age group, educational level, and residence.

These findings are consistent with evidence that pregnancy increases maternal nutritional requirements to support physiological adaptation and fetal development [2]. Micronutrient deficiencies, including deficiencies in iron, folate, iodine, zinc, calcium, and vitamin D, have been associated with adverse maternal and fetal outcomes [7,12]. The WHO recommendations and FIGO guidance therefore emphasize antenatal nutritional counselling and appropriate micronutrient supplementation [3,4].

Suriya et al. similarly reported frequent micronutrient deficiencies and inadequate nutrient intake among pregnant women [13], while Gernand et al. emphasized that micronutrient deficiencies remain an important global maternal- and fetal-health concern [12]. These observations provide clinical context for the association between supplementation level and pregnancy outcome identified in the present study, although the cross-sectional design does not allow a causal relationship to be established.

Diet quality was also associated with pregnancy-related outcomes, although the strength of this relationship differed according to the outcome considered. In the bivariate analysis, women reporting an unbalanced diet had a higher proportion of adverse pregnancy outcomes; however, this association did not remain statistically significant after correction for multiple testing or after multivariable adjustment. In contrast, women reporting an unbalanced diet had 2.33-fold higher adjusted odds of pregnancy complications compared with those reporting a balanced diet (95% CI: 1.26–4.30; *p* = 0.007). The similar direction and magnitude observed in the Firth sensitivity analysis further supported the robustness of this association.

This finding is consistent with the broader evidence summarized by Gernand et al. [12], who described associations between inadequate micronutrient status during pregnancy and several adverse maternal and fetal outcomes. The WHO guidance also recommends nutritional counselling during pregnancy to promote a healthy, balanced diet and adequate gestational weight gain [14], while iron and folic acid supplementation is specifically recommended to reduce maternal iron deficiency and anemia and to support favorable pregnancy outcomes [15]. Direct quantitative comparison with these sources is limited because they evaluate specific nutritional deficiencies or interventions, whereas the present study assessed self-perceived overall diet quality. Therefore, the approximately two-fold higher odds of pregnancy complications observed in our study should be interpreted as an association with perceived diet quality rather than evidence that an unbalanced diet directly caused the reported complications.

Micronutrient supplementation represents another important component of antenatal nutritional care. The WHO states that pregnant women have increased requirements for iron and folic acid, and that daily iron and folic acid supplementation reduces the risk of maternal iron deficiency and anemia [15]. These recommendations are relevant to the present findings, as women reporting no supplementation had substantially higher odds of adverse pregnancy outcome compared with those reporting the use of three or more supplements. Although the observational design of this study does not allow for causal inference, the strong association between supplementation level and pregnancy outcome is consistent with the established importance of adequate micronutrient intake during pregnancy.

Evidence from systematic reviews further supports the potential benefits of multiple-micronutrient supplementation during pregnancy. In the Cochrane review by Keats et al. [16], multiple-micronutrient supplementation was associated with a 12% reduction in the risk of low birth weight (RR = 0.88) and an approximately 8% reduction in the risk of small-for-gestational-age birth (RR = 0.92), while the estimated reduction in preterm birth was more modest (RR = 0.95) [16]. In comparison, the association observed in the present study was substantially larger: women reporting no supplementation had an aOR of 46.15 for adverse pregnancy outcome compared with those reporting three or more supplements, which was attenuated but remained strong in the Firth sensitivity analysis (OR = 37.54; 95% CI: 17.89–85.32). These estimates are not directly comparable because Keats et al. evaluated specific multiple-micronutrient interventions in randomized trials, whereas the present study assessed the self-reported number of supplements and used a composite adverse pregnancy outcome. The markedly larger effect observed in our study should therefore be interpreted cautiously and may partly reflect differences in exposure definition, population characteristics, prenatal care engagement, health literacy, and residual confounding rather than a direct biological effect of the number of supplements used. The magnitude of this estimate may also reflect sparse-data effects within the supplementation categories and should therefore not be interpreted as a precise measure of clinical risk. Although Firth penalization reduced the magnitude of the estimate, the association remained strong, supporting its direction while reinforcing the need for cautious interpretation of its absolute size.

The importance of structured nutritional counselling has also been emphasized through the FIGO Nutrition Checklist. Killeen et al. described the FIGO Nutrition Checklist as a practical tool that can help healthcare professionals identify unbalanced dietary patterns, micronutrient risks, and women who may require further nutritional assessment during pregnancy [17]. The present study supports the practical value of this approach, as both perceived diet quality and supplementation level were associated with pregnancy-related outcomes. Incorporating simple screening tools into prenatal consultations may help identify women at nutritional risk and guide individualized counselling.

Previous abortion history was independently associated with adverse pregnancy outcome in the multivariable analysis. Each additional previous abortion was associated with higher odds of adverse outcome. This finding suggests that reproductive history remains an important factor when interpreting pregnancy outcomes and should be considered a potential risk marker or confounder in future studies. Importantly, the association between supplementation level and pregnancy outcome remained significant even after adjustment for previous abortions, which strengthens the relevance of supplementation as an independent factor in this dataset.

Null and negative findings: Several investigated associations were not statistically significant in the present study. Hand hygiene frequency was not significantly associated with urinary tract infection, and oral hygiene frequency was not significantly associated with diagnosed infection. Similarly, vaccination status was not associated with diagnosed infection, educational level was not significantly associated with the supplementation group, and age group was not significantly associated with adverse pregnancy outcome. These null findings should not be interpreted as evidence that hand hygiene, oral health, vaccination, education, or maternal age are unimportant during pregnancy. Rather, the absence of statistically significant associations may reflect characteristics and limitations of the present sample and measurements.

Statistical power may have been limited for some comparisons, particularly where exposure or outcome groups were small. For example, only a small proportion of participants reported vaccination during pregnancy, limiting the ability to detect anything other than relatively large differences. In addition, the composite outcome of diagnosed infection included heterogeneous infections, many of which are not preventable by the vaccines assessed in this study. Hand and oral hygiene were measured only by self-reported frequency, without information on technique, duration, dental or periodontal status, or other relevant behavioral factors, which may have reduced the sensitivity of these measures. Furthermore, correction for multiple comparisons reduced the likelihood of false-positive findings but also made modest associations more difficult to detect. Therefore, these negative findings should be interpreted cautiously and viewed as inconclusive rather than as evidence against established preventive recommendations or previously reported associations.

Strengths: The present study has several strengths. It evaluated hygiene- and nutrition-related behaviors simultaneously in relation to multiple clinically relevant outcomes, including diagnosed infections, pregnancy complications, and adverse pregnancy outcomes. The analysis combined descriptive, bivariate, and multivariable approaches, allowing for adjustment for several potential confounders, including age group, educational level, residence, reproductive history, diet quality, supplementation level, and hygiene-related variables. The use of Bonferroni correction for predefined bivariate analyses reduced the risk of false-positive findings due to multiple testing, while multicollinearity assessment and Firth’s penalized logistic regression sensitivity analyses provided additional checks on the robustness of the multivariable results.

Limitations: Nevertheless, several limitations must be acknowledged. Key measurement limitations included the subjective self-assessment of diet quality, the lack of detailed information on intimate hygiene products, the absence of data on sexual activity, and the lack of information on gestational age at questionnaire completion. First, the cross-sectional observational design does not allow temporal relationships or causality to be established, and reverse causation cannot be excluded. Second, the data was self-reported, which may have introduced recall and reporting bias, particularly for previous pregnancy outcomes, infections, supplementation, and hygiene behaviors. Some outcomes, including vaginal candidiasis and vaccination status, involved relatively small subgroups, resulting in wider confidence intervals and reduced precision. In addition, diet quality was assessed subjectively rather than using a validated dietary assessment instrument, and hygiene behaviors were assessed primarily by reported frequency rather than by detailed evaluation of technique or products used. More than that, nutritional supplementation was categorized according to the number of reported supplements rather than their specific composition, dose, duration of use, or clinical indication. Therefore, this variable should be interpreted as a simplified measure of supplementation behavior rather than as a direct measure of nutritional adequacy or the effect of individual supplements. The questionnaire did not explicitly distinguish whether the reported number of abortions referred exclusively to pregnancy losses preceding the most recent pregnancy. Therefore, some overlap between abortion history and the composite adverse pregnancy outcome cannot be excluded, and the corresponding regression estimate should be interpreted cautiously.

Several potentially important confounders were not measured, including socioeconomic status, pre-pregnancy and gestational body mass index, smoking status, gestational age at questionnaire completion, prenatal care utilization, previous specific adverse pregnancy outcomes such as preterm birth, chronic hypertension, pregestational diabetes mellitus, chronic cardiovascular or respiratory disease, sexual activity, vaginal douching, use of intimate hygiene products, and other relevant maternal comorbidities. Socioeconomic status and prenatal care utilization may be associated with both greater use of nutritional supplements and more favorable pregnancy outcomes; consequently, failure to adjust for these factors could have contributed to an overestimation of the association between supplementation level and pregnancy outcome. Similarly, smoking and pre-pregnancy body mass index may be related to both diet quality and pregnancy complications and could either strengthen or attenuate the observed dietary associations depending on their distribution in the study population. Sexual activity, previous urinary tract infection, vaginal douching, and the use of intimate hygiene products may independently influence the risk of genital or urinary infection while also being related to reported hygiene behaviors; therefore, residual confounding may partly explain the observed associations between intimate hygiene frequency and infection-related outcomes. Finally, gestational age at questionnaire completion may have resulted in different periods of opportunity for exposure to supplementation and for the occurrence of infections or complications. These potential sources of residual confounding should be considered when interpreting the magnitude of the reported associations. Because these variables were not collected in the original questionnaire, they could not be retrospectively incorporated into the multivariable models. Their omission therefore represents an important source of potential residual confounding and limits the interpretation of the reported adjusted associations as fully independent effects.

Taken together, the present findings indicate that the strongest associations were observed for intimate hygiene frequency, nutritional supplementation, diet quality, and previous abortion history. The sensitivity analyses supported the overall robustness of the principal multivariable findings, although the magnitude of some estimates, particularly the association between supplementation and pregnancy outcome, warrants cautious interpretation. The cross-sectional design, self-reported exposures, and potential residual confounding prevent causal inference. Accordingly, the results should be viewed as hypothesis-generating evidence supporting more detailed prospective investigation of hygiene and nutritional behaviors during pregnancy.

## 5. Conclusions

In this cross-sectional study, self-reported nutritional supplementation, perceived diet quality, intimate hygiene frequency, and reproductive history were associated with selected pregnancy-related outcomes. The strongest associations were observed between lower levels of reported supplementation and adverse pregnancy outcome, between occasional intimate hygiene and infection-related outcomes, and between perceived diet quality and pregnancy complications. The additional sensitivity analyses generally supported the persistence of the principal associations, although some estimates were attenuated and became less precise in restricted samples.

These findings should be interpreted cautiously and regarded as hypothesis-generating rather than as evidence of causal or preventive effects. The cross-sectional design, reliance on self-reported exposures, lack of information on several potentially important confounders, and uncertainty regarding the temporal relationship between some exposures and pregnancy outcomes limit causal interpretation. In particular, the observed associations should not be interpreted as demonstrating that a specific supplementation level or hygiene frequency directly prevents adverse outcomes.

Prospective studies are needed to confirm these observations using objective or validated assessments of nutritional intake and hygiene practices, detailed information on the composition, dose, duration, and timing of supplementation, assessment of relevant behavioral and clinical confounders, and clinical confirmation of pregnancy-related outcomes. Such studies would help determine whether the associations observed here are reproducible and clinically meaningful and would provide a stronger basis for future preventive recommendations.

## Figures and Tables

**Table 1:** General characteristics of the study group.

Parameter	Category	Total, *n* (%)	Term Outcome, *n* (%)	Adverse Outcome, *n* (%)	χ^2^ (df)	*p*-Value
Age	18–25 years	46 (12.7)	33 (12.5)	13 (13.4)	3.72 (3)	0.294
	26–30 years	127 (35.2)	99 (37.5)	28 (28.9)		
	31–35 years	65 (18.0)	49 (18.6)	16 (16.5)		
	36–40 years	123 (34.1)	83 (31.4)	40 (41.2)		
Area of residence	Urban	260 (72.0)	196 (74.2)	64 (66.0)	2.01 (1)	0.156
	Rural	101 (28.0)	68 (25.8)	33 (34.0)		
Educational level	High school	111 (30.7)	77 (29.2)	34 (35.1)	3.50 (2)	0.174
	University studies	206 (57.1)	150 (56.8)	56 (57.7)		
	Postgraduate studies	44 (12.2)	37 (14.0)	7 (7.2)		
Reproductive status	Previous pregnancy ending in birth	222 (61.5)	174 (65.9)	48 (49.5)	8.80 (2)	0.012
	Currently pregnant	102 (28.3)	68 (25.8)	34 (35.1)		
	Previous spontaneous abortion	37 (10.2)	22 (8.3)	15 (15.5)		
Number of previous abortions	0	186 (51.5)	161 (61.0)	25 (25.8)	36.67 (3)	<0.001
	1	156 (43.2)	90 (34.1)	66 (68.0)		
	2	14 (3.9)	9 (3.4)	5 (5.2)		
	3	5 (1.4)	4 (1.5)	1 (1.0)		

Data is presented as *n* (%). Percentages in the term- and adverse-outcome columns are calculated within each outcome group. Adverse pregnancy outcome included spontaneous abortion, preterm birth, and post-term pregnancy. Chi-square tests were used to compare categorical distributions between outcome groups. The reproductive-status category “previous spontaneous abortion” is distinct from the variable “number of previous abortions.” The latter records abortion history across all reproductive-status categories; therefore, participants classified as currently pregnant or as having a previous pregnancy ending in birth could also report one or more previous abortions.

**Table 2:** Obstetric and medical characteristics according to pregnancy outcome.

Parameter	Category	Total, *n* (%)	Term Outcome, *n* (%)	Adverse Outcome, *n* (%)	Statistic Test	*p*-Value
Outcome of the most recent pregnancy	Term delivery	264 (73.1)	264 (100.0)	0 (0.0)	Not applicable	Not applicable
	Spontaneous abortion	55 (15.2)	0 (0.0)	55 (56.7)		
	Preterm delivery (<37 weeks)	38 (10.5)	0 (0.0)	38 (39.2)		
	Post-term pregnancy (>41 weeks)	4 (1.1)	0 (0.0)	4 (4.1)		
Pregnancy complications or infections	No	188 (52.1)	146 (55.3)	42 (43.3)	χ^2^ = 4.10; df = 1	0.043
	Yes	173 (47.9)	118 (44.7)	55 (56.7)		
Diagnosed infection during pregnancy	No	270 (74.8)	204 (77.3)	66 (68.0)	χ^2^ = 3.21; df = 1	0.073
	Yes	91 (25.2)	60 (22.7)	31 (32.0)		
Vaccination during pregnancy	Not vaccinated/unknown	342 (94.7)	250 (94.7)	92 (94.8)	Fisher’s exact test	1.000
	Vaccinated	19 (5.3)	14 (5.3)	5 (5.2)		

Data is presented as *n* (%). Percentages in the term- and adverse-outcome columns are calculated within each outcome group. The adverse pregnancy outcome group comprised spontaneous abortion, preterm delivery, and post-term pregnancy. For the rows describing the outcome of the most recent pregnancy, inferential testing was not applicable because these categories define the term and adverse outcome groups. Pearson’s Chi-square test was used for categorical comparisons, while Fisher’s exact test was used when expected cell frequencies were small.

**Table 3:** Hygiene- and nutrition-related behaviors according to pregnancy outcome.

Parameter	Category	Total, *n* (%)	Term Outcome, *n* (%)	Adverse Outcome, *n* (%)	χ^2^ (df)	*p*-Value
Supplementation during pregnancy	No supplements	71 (19.7)	13 (4.9)	58 (59.8)	142.22 (2)	<0.001
	1–2 supplements	59 (16.3)	43 (16.3)	16 (16.5)		
	≥3 supplements	231 (64.0)	208 (78.8)	23 (23.7)		
Frequency of hand washing	<3 times/day	69 (19.1)	46 (17.4)	23 (23.7)	2.02 (2)	0.365
	3–5 times/day	163 (45.2)	120 (45.5)	43 (44.3)		
	6–10 times/day	129 (35.7)	98 (37.1)	31 (32.0)		
Oral hygiene	Once/day	81 (22.4)	51 (19.3)	30 (30.9)	5.68 (2)	0.058
	Twice/day	242 (67.0)	183 (69.3)	59 (60.8)		
	Irregularly	38 (10.5)	30 (11.4)	8 (8.2)		
Intimate hygiene	Occasionally	59 (16.3)	35 (13.3)	24 (24.7)	6.86 (2)	0.032
	Once/day	188 (52.1)	143 (54.2)	45 (46.4)		
	Twice/day	114 (31.6)	86 (32.6)	28 (28.9)		
Perceived diet quality	Unbalanced	70 (19.4)	43 (16.3)	27 (27.8)	6.38 (2)	0.041
	Relatively balanced	149 (41.3)	111 (42.0)	38 (39.2)		
	Balanced	142 (39.3)	110 (41.7)	32 (33.0)		

Data is presented as *n* (%). Percentages in the term and adverse outcome columns were calculated within each outcome group. Adverse pregnancy outcome included spontaneous abortion, preterm birth, and post-term pregnancy. Pearson’s Chi-square test was used to compare categorical distributions between outcome groups.

**Table 4:** Bivariate analysis, main findings.

Association Tested	Test	Statistic	Raw *p*-Value	Bonferroni-Adjusted *p*-Value	Interpretation
Intimate hygiene × vaginal candidiasis	Chi-square	χ^2^ = 46.10; df = 2	<0.001	<0.001	Significant
Hand hygiene × urinary tract infection	Chi-square	χ^2^ = 5.71; df = 2	0.058	0.345	Not significant
Intimate hygiene × urinary tract infection	Chi-square	χ^2^ = 11.48; df = 2	0.003	0.019	Significant after *p*-adjustment
Oral hygiene × diagnosed infection	Chi-square	χ^2^ = 2.35; df = 2	0.309	1.000	Not significant
Diet quality × adverse pregnancy outcome	Chi-square	χ^2^ = 6.38; df = 2	0.041	0.247	Not significant after correction
Supplementation group × pregnancy outcome	Chi-square	χ^2^ = 142.22; df = 2	<0.001	<0.001	Significant

**Table 5:** Bivariate analysis, secondary findings.

Association Tested	Test	Statistic	Raw *p*-Value	Bonferroni-Adjusted *p*-Value	Interpretation
Vaccination status × diagnosed infection	Fisher’s exact test	-	0.424	1.000	Not significant
Educational level × supplementation group	Chi-square	χ^2^ = 7.61; df = 4	0.107	0.321	Not significant
Age group × adverse pregnancy outcome	Chi-square	χ^2^ = 3.72; df = 3	0.294	0.882	Not significant

**Table 6:** Multivariable logistic regression analysis.

Model/Outcome	Significant or Relevant Predictor	Adjusted OR	95% CI	*p*-Value
Model 1: Adverse pregnancy outcome	1–2 supplements vs. ≥3 supplements	3.32	1.52–7.21	0.002
	No supplements vs. ≥3 supplements	46.15	20.45–104.15	<0.001
	Number of previous abortions	2.25	1.41–3.59	<0.001
	Unbalanced diet vs. balanced diet	2.11	0.90–4.94	0.085
Model 2: Any pregnancy complication	Unbalanced diet vs. balanced diet	2.33	1.26–4.30	0.007
	Relatively balanced diet vs. balanced diet	1.63	0.99–2.66	0.052
	Occasional intimate hygiene vs. twice/day	2.39	1.21–4.72	0.012
Model 3: Any diagnosed infection	Occasional intimate hygiene vs. twice/day	5.01	2.34–10.71	<0.001
	Relatively balanced diet vs. balanced diet	0.51	0.28–0.92	0.025
	Vaccination	0.59	0.16–2.14	0.421
Model 4: Vaginal candidiasis	Occasional intimate hygiene vs. twice/day	6.63	2.29–19.17	<0.001
	Once/day intimate hygiene vs. twice/day	0.31	0.07–1.28	0.105
Model 5: Urinary tract infection	Occasional intimate hygiene vs. twice/day	4.36	1.73–10.99	0.002
	Once/day intimate hygiene vs. twice/day	2.35	1.04–5.29	0.040
	Total number of pregnancies	1.46	1.08–1.98	0.013

The table presents estimates from conventional multivariable logistic regression. Firth’s penalized logistic regression was additionally performed as a sensitivity analysis; the principal findings remained materially unchanged, as described in the Results section. aOR, adjusted odds ratio; CI, confidence interval.

**Table 7:** Diagnostic performance of the multivariable logistic regression models.

Model	Outcome	Observations, *n*	Outcome Events, *n*	AUC
1	Adverse pregnancy outcome	361	97	0.862
2	Any pregnancy complication or infection	361	173	0.650
3	Any diagnosed infection	361	91	0.692
4	Vaginal candidiasis	361	25	0.832
5	Urinary tract infection	361	53	0.696

AUC, area under the receiver operating characteristic curve. All models included the complete analytical sample because no missing values were present for the variables entered into the respective models.

## Data Availability

The collected data cannot be shared as it contradicts the informed consent that participants agreed to. Further inquiries can be directed to the corresponding authors.

## Supplementary Materials

The following supporting information can be downloaded at: https://www.mdpi.com/article/10.3390/medicina62091676/s1. File S1: English Translation of the Study Questionnaire; File S2: ROC discrimination and calibration of the multivariable logistic regression models; File S3: Complete conventional and Firth penalized logistic regression results.

## Abbreviations

The following abbreviations are used in this manuscript:

aOR	Adjusted odds ratio
CI	Confidence interval
CMV	Cytomegalovirus
COVID-19	Coronavirus disease 2019
df	Degrees of freedom
FIGO	International Federation of Gynecology and Obstetrics
OR	Odds ratio
WHO	World Health Organization
χ^2^	Chi-square statistic
