# Supplementary material for: Associations Between Hygiene- and Nutrition-Related Practices and Pregnancy Outcomes: A Cross-Sectional Study
Source: Medicina (Kaunas). 2026 Aug 31;62(9):1676. doi: 10.3390/medicina62091676 (PMC13609565; doi:10.3390/medicina62091676)
Supplement: Supplementary file 1 [file medicina-62-01676-s001.zip › Supplementary Material File S1.pdf]

Supplementary Material S1

English Translation of the Study Questionnaire

Reconstructed from the original Romanian questionnaire export used for the study. The consent item is shown separately from the 16 study items. Question wording follows the exported column headings. For categorical items, response options are based on the exported responses; bacterial vaginosis is additionally retained because it is explicitly listed among the questionnaire response categories in the manuscript Methods. Options that were never selected cannot be independently recovered from the response export.

No.	Question	Response type	Response options
1	Do you agree to participate?	Consent item	Yes
2	Age	Single choice	• 18–25 years<br>• 26–30 years<br>• 31–35 years<br>• 36–40 years
3	Area of residence	Single choice	• Urban<br>• Rural
4	Educational level	Single choice	• High school<br>• University studies<br>• Postgraduate studies
5	Reproductive status	Single choice	• Currently pregnant<br>• I have had a pregnancy ending in birth<br>• I have had a spontaneous abortion
6	Total number of pregnancies	Numeric response	Numeric entry
7	Number of births	Numeric response	Numeric entry
8	Number of abortions	Numeric response	Numeric entry
9	Outcome of the most recent pregnancy	Single choice	• Term birth<br>• Preterm birth (<37 weeks)<br>• Post-term pregnancy (>41 weeks)<br>• Spontaneous abortion
10	Did you have complications or infections during pregnancy?	Multiple selections observed	• No complications<br>• Urinary tract infection<br>• Vaginal candidiasis<br>• Bacterial vaginosis*<br>• Influenza<br>• Cytomegalovirus (CMV) infection<br>• Toxoplasmosis<br>• Anemia<br>• Gestational diabetes<br>• Hypertension during pregnancy
11	Did you use any of the following supplements during pregnancy?	Multiple selections observed	• Folic acid<br>• Vitamin D<br>• Iron<br>• Magnesium<br>• Vitamin C<br>• Vitamin B6<br>• Vitamin B12<br>• Zinc<br>• Vitamin E<br>• I did not use supplements
12	Were you diagnosed during pregnancy with any of the following?	Multiple selections observed	• None<br>• Urinary tract infection<br>• Vaginal candidiasis<br>• Bacterial vaginosis*<br>• Influenza<br>• Cytomegalovirus (CMV) infection<br>• Toxoplasmosis
13	Did you receive any vaccinations during pregnancy?	Categorical response	• No<br>• I do not know<br>• COVID-19 vaccine<br>• Influenza vaccine
14	How often do you wash your hands each day?	Single choice	• <3 times/day<br>• 3–5 times/day<br>• 6–10 times/day
15	How many times per day do you brush your teeth?	Single choice	• Once/day<br>• Twice/day<br>• Irregularly

16	How often do you perform intimate hygiene?	Single choice	• Occasionally<br>• Once/day<br>• Twice/day
17	How would you describe your diet during pregnancy?	Single choice	• Unbalanced<br>• Relatively balanced<br>• Balanced

** Bacterial vaginosis is listed in the manuscript as a questionnaire response category but does not appear among the selected responses in the exported dataset.*

Note on Questions 14–17: the exported analytical dataset contains one response category per participant for each of these four items, consistent with mutually exclusive single-choice responses.
