# Supplementary material for: Associations Between Hygiene- and Nutrition-Related Practices and Pregnancy Outcomes: A Cross-Sectional Study
Source: Medicina (Kaunas). 2026 Aug 31;62(9):1676. doi: 10.3390/medicina62091676 (PMC13609565; doi:10.3390/medicina62091676)
Supplement: Supplementary file 1 [file medicina-62-01676-s001.zip › Supplementary Material File S2.pdf]

Supplementary Material 2

ROC discrimination and calibration of the multivariable logistic regression models

Model discrimination was assessed using receiver operating characteristic (ROC) curves and the area under the ROC curve (AUC). Calibration was evaluated by sorting participants according to predicted probability and dividing them into five approximately equal-sized groups. Each calibration point represents the mean predicted probability and the observed outcome proportion within one group; the diagonal line represents perfect calibration. No missing values were present in the variables included in the five models, so all models included the complete analytical sample of 361 participants.

Table S2.1. Diagnostic indicators for the five multivariable logistic regression models.

Model	Outcome	Observations, n	Outcome events, n	AUC
1	Adverse pregnancy outcome	361	97	0.862
2	Any pregnancy complication or infection	361	173	0.650
3	Any diagnosed infection	361	91	0.692
4	Vaginal candidiasis	361	25	0.832
5	Urinary tract infection	361	53	0.696

AUC, area under the receiver operating characteristic curve. All models used n = 361 observations because no missing values were present in the variables entered into the respective models.

Model 1: Adverse pregnancy outcome

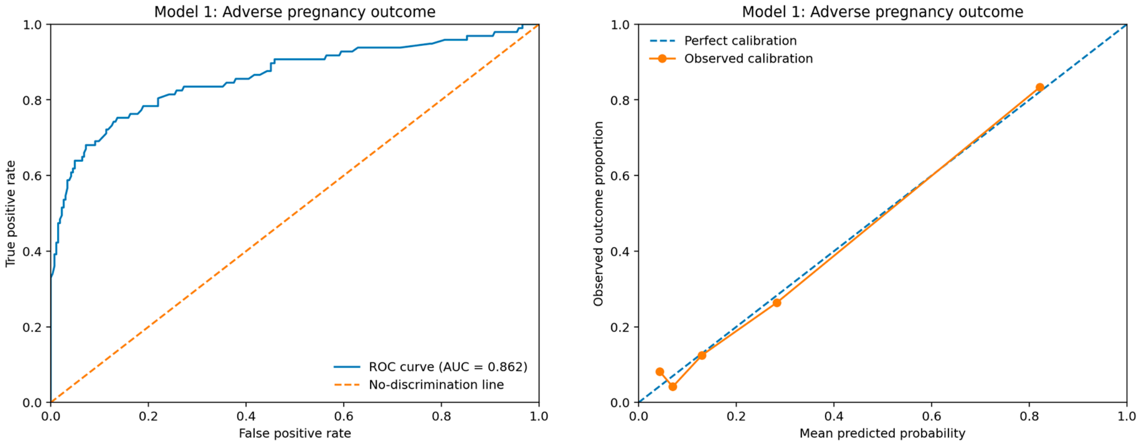

Figure S2.1. ROC curve for Model 1 (Adverse pregnancy outcome). n = 361; outcome events = 97; AUC = 0.862. The dashed diagonal line represents no discrimination.

Figure S2.2. Calibration plot for Model 1 (Adverse pregnancy outcome). n = 361; outcome events = 97. Points represent observed outcome proportions versus mean predicted probabilities across five approximately equal-sized groups of predicted risk; the dashed diagonal line indicates perfect calibration.

Model 2: Any pregnancy complication or infection

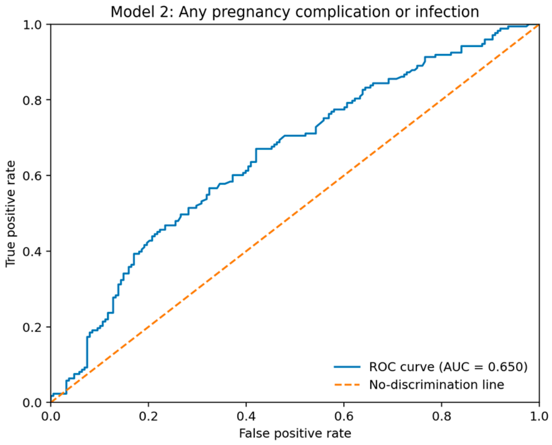

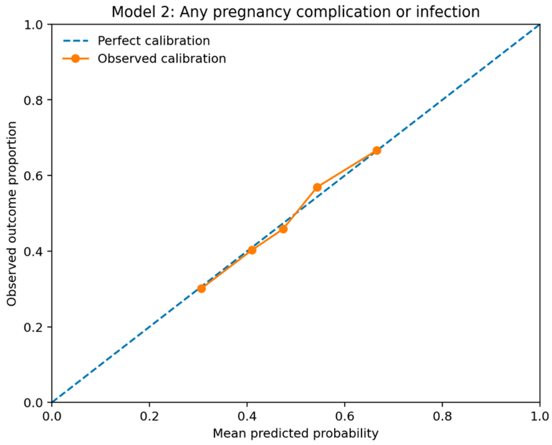

Figure S2.3. ROC curve for Model 2 (Any pregnancy complication or infection). n = 361; outcome events = 173; AUC = 0.650. The dashed diagonal line represents no discrimination.

Figure S2.4. Calibration plot for Model 2 (Any pregnancy complication or infection). n = 361; outcome events = 173. Points represent observed outcome proportions versus mean predicted probabilities across five approximately equal-sized groups of predicted risk; the dashed diagonal line indicates perfect calibration.

Model 3: Any diagnosed infection

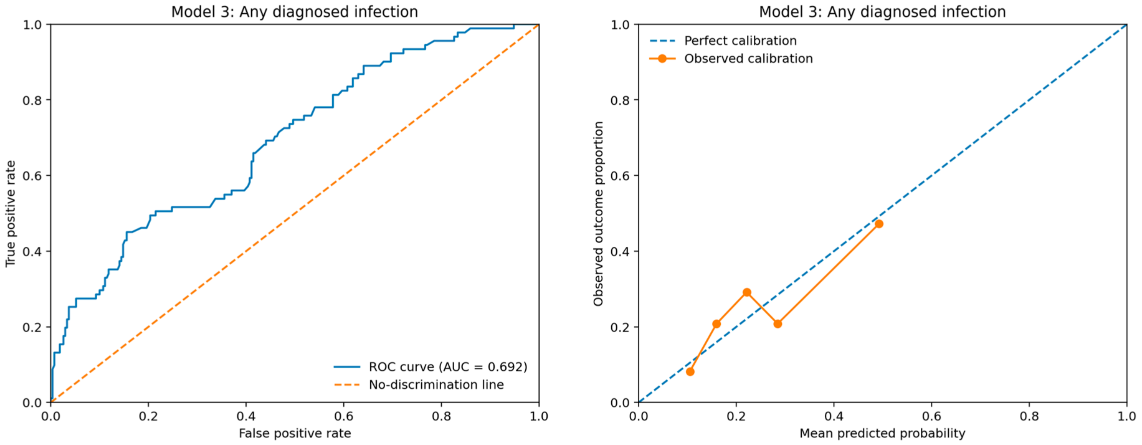

Figure S2.5. ROC curve for Model 3 (Any diagnosed infection). n = 361; outcome events = 91; AUC = 0.692. The dashed diagonal line represents no discrimination.

Figure S2.6. Calibration plot for Model 3 (Any diagnosed infection). n = 361; outcome events = 91. Points represent observed outcome proportions versus mean predicted probabilities across five approximately equal-sized groups of predicted risk; the dashed diagonal line indicates perfect calibration.

Model 4: Vaginal candidiasis

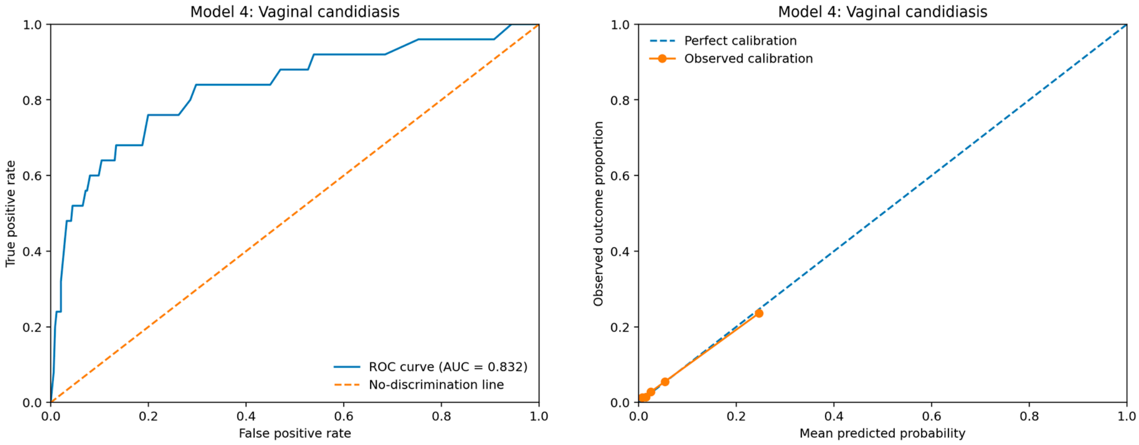

Figure S2.7. ROC curve for Model 4 (Vaginal candidiasis). n = 361; outcome events = 25; AUC = 0.832. The dashed diagonal line represents no discrimination.

Figure S2.8. Calibration plot for Model 4 (Vaginal candidiasis). n = 361; outcome events = 25. Points represent observed outcome proportions versus mean predicted probabilities across five approximately equal-sized groups of predicted risk; the dashed diagonal line indicates perfect calibration.

Model 5: Urinary tract infection

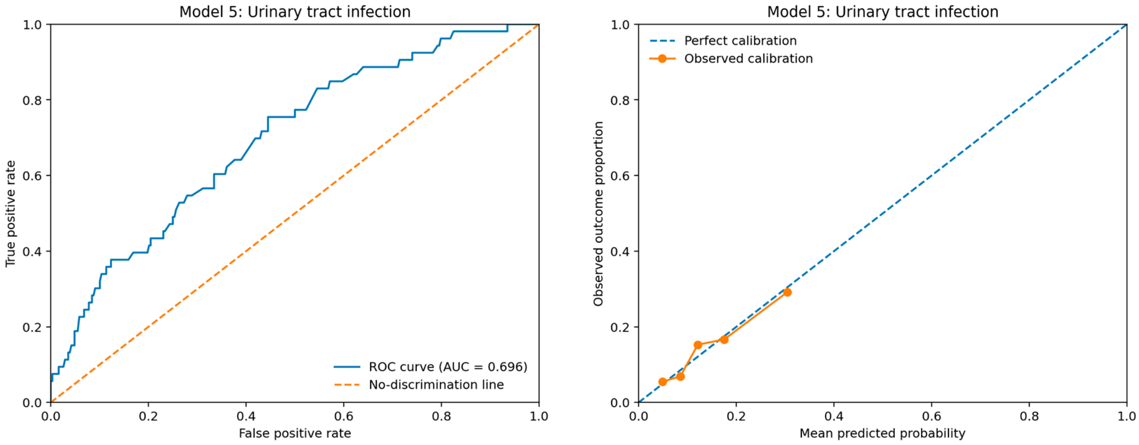

Figure S2.9. ROC curve for Model 5 (Urinary tract infection). n = 361; outcome events = 53; AUC = 0.696. The dashed diagonal line represents no discrimination.

Figure S2.10. Calibration plot for Model 5 (Urinary tract infection). n = 361; outcome events = 53. Points represent observed outcome proportions versus mean predicted probabilities across five approximately equal-sized groups of predicted risk; the dashed diagonal line indicates perfect calibration.
