# Supplementary material for: Associations Between Hygiene- and Nutrition-Related Practices and Pregnancy Outcomes: A Cross-Sectional Study
Source: Medicina (Kaunas). 2026 Aug 31;62(9):1676. doi: 10.3390/medicina62091676 (PMC13609565; doi:10.3390/medicina62091676)
Supplement: Supplementary file 1 [file medicina-62-01676-s001.zip › Supplementary Material File S3.pdf]

Supplementary Material 3

Complete conventional and Firth penalized logistic regression results

Complete coefficient estimates for all predictor levels entered into the five multivariable models are presented below. All models used the complete analytical sample of 361 participants. Intercepts are omitted for readability.

Table S3.1. Model 1: Adverse pregnancy outcome

Predictor / comparison	Conventional aOR	95% CI	p-value	Firth OR	95% CI	p-value
1–2 supplements vs ≥3 supplements	3.32	1.52–7.21	0.002	3.16	1.48–6.73	0.003
No supplements vs ≥3 supplements	46.15	20.45–104.15	<0.001	37.54	17.89–85.32	<0.001
Relatively balanced diet vs balanced diet	0.96	0.46–2.01	0.913	0.96	0.47–1.98	0.918
Unbalanced diet vs balanced diet	2.11	0.90–4.94	0.085	2.05	0.89–4.70	0.091
Age 26–30 vs 18–25 years	0.70	0.25–1.97	0.501	0.70	0.26–1.96	0.486
Age 36–40 vs 18–25 years	0.85	0.31–2.37	0.762	0.85	0.32–2.35	0.746
Age 31–35 vs 18–25 years	0.66	0.21–2.06	0.472	0.67	0.22–2.05	0.478
University education vs high school	1.38	0.66–2.87	0.392	1.35	0.67–2.81	0.409
Postgraduate education vs high school	0.46	0.14–1.57	0.217	0.50	0.15–1.56	0.238
Rural vs urban residence	1.28	0.64–2.58	0.483	1.27	0.64–2.50	0.490
Number of previous abortions (per additional abortion)	2.25	1.41–3.59	<0.001	2.16	1.38–3.43	<0.001

Note: aOR, adjusted odds ratio; CI, confidence interval. Conventional estimates are from standard multivariable binary logistic regression. Firth estimates are from penalized-likelihood logistic regression; profile penalized-likelihood confidence intervals are reported. Reference categories are stated within each comparison.

Table S3.2. Model 2: Any pregnancy complication or infection

Predictor / comparison	Conventional aOR	95% CI	p-value	Firth OR	95% CI	p-value
Relatively balanced diet vs balanced diet	1.63	0.99–2.66	0.052	1.59	0.99–2.59	0.057
Unbalanced diet vs balanced diet	2.33	1.26–4.30	0.007	2.23	1.23–4.10	0.008
1–2 supplements vs ≥3 supplements	0.55	0.30–1.03	0.063	0.57	0.31–1.04	0.067
No supplements vs ≥3 supplements	1.08	0.61–1.92	0.788	1.08	0.62–1.89	0.794
Intimate hygiene once/day vs twice/day	1.26	0.77–2.08	0.353	1.25	0.77–2.04	0.366
Occasional intimate hygiene vs twice/day	2.39	1.21–4.72	0.012	2.29	1.19–4.48	0.013
Oral hygiene once/day vs twice/day	0.96	0.56–1.65	0.887	0.96	0.57–1.63	0.893
Irregular oral hygiene vs twice/day	1.77	0.86–3.64	0.122	1.71	0.86–3.50	0.129
Age 26–30 vs 18–25 years	1.37	0.67–2.84	0.391	1.35	0.67–2.77	0.402
Age 36–40 vs 18–25 years	1.10	0.53–2.29	0.802	1.09	0.53–2.25	0.810
Age 31–35 vs 18–25 years	0.94	0.42–2.11	0.886	0.94	0.43–2.07	0.886
University education vs high school	1.14	0.70–1.86	0.604	1.13	0.70–1.83	0.612
Postgraduate education vs high school	0.61	0.29–1.30	0.202	0.63	0.30–1.30	0.213
Rural vs urban residence	1.20	0.74–1.96	0.462	1.19	0.74–1.93	0.474
Total number of pregnancies (per additional pregnancy)	1.07	0.85–1.34	0.578	1.06	0.85–1.33	0.590

Note: aOR, adjusted odds ratio; CI, confidence interval. Conventional estimates are from standard multivariable binary logistic regression. Firth estimates are from penalized-likelihood logistic regression; profile penalized-likelihood confidence intervals are reported. Reference categories are stated within each comparison.

Table S3.3. Model 3: Any diagnosed infection

Predictor / comparison	Conventional aOR	95% CI	p-value	Firth OR	95% CI	p-value
Intimate hygiene once/day vs twice/day	1.55	0.83–2.90	0.166	1.51	0.83–2.82	0.177
Occasional intimate hygiene vs twice/day	5.01	2.34–10.71	<0.001	4.59	2.22–9.75	<0.001
Hand washing 3–5 times/day vs 6–10 times/day	0.68	0.38–1.22	0.194	0.69	0.39–1.22	0.202
Hand washing <3 times/day vs 6–10 times/day	1.48	0.74–2.94	0.264	1.45	0.74–2.83	0.273
Relatively balanced diet vs balanced diet	0.51	0.28–0.92	0.025	0.52	0.29–0.93	0.027
Unbalanced diet vs balanced diet	1.03	0.52–2.04	0.936	1.03	0.53–2.00	0.919
1–2 supplements vs ≥3 supplements	0.82	0.39–1.73	0.603	0.84	0.40–1.70	0.638
No supplements vs ≥3 supplements	1.00	0.52–1.94	0.994	1.01	0.53–1.89	0.970
University education vs high school	0.77	0.43–1.37	0.366	0.77	0.44–1.37	0.374
Postgraduate education vs high school	0.67	0.27–1.62	0.371	0.70	0.28–1.61	0.406
Rural vs urban residence	1.48	0.84–2.58	0.172	1.45	0.84–2.49	0.181
Age 26–30 vs 18–25 years	0.94	0.42–2.09	0.882	0.93	0.44–2.06	0.863
Age 36–40 vs 18–25 years	0.75	0.33–1.72	0.495	0.75	0.34–1.71	0.492
Age 31–35 vs 18–25 years	0.57	0.23–1.43	0.231	0.59	0.24–1.43	0.241
Vaccinated vs not vaccinated/unknown	0.59	0.16–2.14	0.421	0.67	0.17–1.99	0.493

Note: aOR, adjusted odds ratio; CI, confidence interval. Conventional estimates are from standard multivariable binary logistic regression. Firth estimates are from penalized-likelihood logistic regression; profile penalized-likelihood confidence intervals are reported. Reference categories are stated within each comparison.

Table S3.4. Model 4: Vaginal candidiasis

Predictor / comparison	Conventional aOR	95% CI	p-value	Firth OR	95% CI	p-value
Intimate hygiene once/day vs twice/day	0.31	0.07–1.28	0.105	0.34	0.08–1.23	0.100
Occasional intimate hygiene vs twice/day	6.63	2.29–19.17	<0.001	5.73	2.18–16.77	<0.001
Relatively balanced diet vs balanced diet	0.52	0.18–1.50	0.227	0.55	0.19–1.48	0.237
Unbalanced diet vs balanced diet	1.02	0.32–3.25	0.978	1.04	0.33–3.10	0.942
University education vs high school	0.46	0.16–1.31	0.147	0.49	0.18–1.32	0.155
Postgraduate education vs high school	1.46	0.41–5.13	0.557	1.48	0.43–4.80	0.519
Age 26–30 vs 18–25 years	0.73	0.20–2.59	0.621	0.71	0.22–2.53	0.591
Age 36–40 vs 18–25 years	0.41	0.09–1.85	0.248	0.44	0.10–1.81	0.251
Age 31–35 vs 18–25 years	0.93	0.23–3.71	0.915	0.92	0.24–3.55	0.905

Note: aOR, adjusted odds ratio; CI, confidence interval. Conventional estimates are from standard multivariable binary logistic regression. Firth estimates are from penalized-likelihood logistic regression; profile penalized-likelihood confidence intervals are reported. Reference categories are stated within each comparison.

Table S3.5. Model 5: Urinary tract infection

Predictor / comparison	Conventional aOR	95% CI	p-value	Firth OR	95% CI	p-value
Intimate hygiene once/day vs twice/day	2.35	1.04–5.29	0.040	2.21	1.04–5.11	0.039
Occasional intimate hygiene vs twice/day	4.36	1.73–10.99	0.002	4.04	1.68–10.20	0.002
Hand washing 3–5 times/day vs 6–10 times/day	0.63	0.31–1.28	0.201	0.64	0.32–1.28	0.205
Hand washing <3 times/day vs 6–10 times/day	1.39	0.63–3.08	0.416	1.38	0.63–2.97	0.421
Age 26–30 vs 18–25 years	0.81	0.32–2.02	0.649	0.80	0.33–2.00	0.621
Age 36–40 vs 18–25 years	0.63	0.24–1.66	0.353	0.64	0.25–1.66	0.346
Age 31–35 vs 18–25 years	0.49	0.17–1.45	0.197	0.51	0.18–1.44	0.203
University education vs high school	1.04	0.53–2.05	0.902	1.03	0.54–2.04	0.921
Postgraduate education vs high school	0.55	0.17–1.81	0.323	0.60	0.17–1.75	0.365
Total number of pregnancies (per additional pregnancy)	1.46	1.08–1.98	0.013	1.44	1.07–1.94	0.015

Note: aOR, adjusted odds ratio; CI, confidence interval. Conventional estimates are from standard multivariable binary logistic regression. Firth estimates are from penalized-likelihood logistic regression; profile penalized-likelihood confidence intervals are reported. Reference categories are stated within each comparison.
